# Our Contemporaries

**Published:** 1913-12

**Authors:** 


### OUR CONTEMPORARIES

The *Medical Review,* St. Louis, September, 1913, contains an article by Dr. John D. Bonnar of Buffalo on "Medicine as a Science."

Dr. Edward C. Hill has resigned the editorship of the *Denver Medical Times* just because he is 50 years old and had promised himself long ago to limit his attention to practice after that age. We hope to continue the management of this JOURNAL through Vol 100, which will bring us somewhat past that age.

The *Caledonian Medical Journal* of October, 1913, contains an interesting biography of Dr. David Livingston, who was born 100 years ago. Let us not forget that this great man, distinguished as an African explorer, was also a physician.

*Nevada Medicine* (published jointly with the *Denver Medical Times* and the *Utah Medical Journal*) calls attention to the newly established American College of Surgeons, which has just met in Chicago, and condemns the proposal to secure legislation restricting the performance of major operations to this body. The argument is based partly on the contention that 5,000 surgeons cannot do the surgical work of the country and partly on general principles of vested professional rights and democratic ideals. While one surgeon should, on the average, be sufficient for the surgical part of twenty-five average general practices, it is obvious that surgeons cannot always be properly distributed nor available. There are also obvious difficulties in drawing formal lines between the conditions requiring expert surgical skill and those properly left to the general practitioner and in establishing conditions of emergency. Granting that the College was absolutely disinterested and just in its admissions, there would still be a serious difficulty in withdrawing the general licenses to practice all branches of medicine, already in force. It would be half a century before these general licenses would lapse by death, and it is entirely possible that various changes might occur before that time which would render any such limitation of practice highly undesirable. Meantime, it would be unwise and unjust to discriminate between practitioners simply on account of the holding over of a right conferred under the present system of license. We would naturally be prejudiced in favor of requiring special license for specialized lines of medical work, but the more one looks into the matter the more difficult of solution do the problems involved in such schemes become. The present natural development by which each specialist, surgical or otherwise, must make good as an individual, by which every practitioner is guided by his own conscience in regard to what he can and what he cannot properly undertake, and by which each patient must assume the responsibility of a wise choice of attendant, works well in the vast majority of instances, and we doubt very much whether any artificial system can improve upon it. At any rate, until the American College of Surgeons has established its right to control surgery by some years of successful existence and has done away with the prejudice quoted by actual administration of its affairs in a way satisfactory to the profession generally, it is premature even to announce any such radical change of medical licensure as is suggested. All of which does not by any means imply either personal or editorial prejudice against this body nor any lack of hope for its future.

---

The *American Journal of Surgery* will issue a special "Fracture Number" in January, containing articles by prominent surgeons of this country. The territory of the BUFFALO MEDICAL JOURNAL will be represented by Dr. E. S. Van Duyn of Syracuse.

